# Testing spatial transferability of species distribution models reveals differing habitat preferences for an endangered delphinid (*Cephalorhynchus hectori*) in Aotearoa, New Zealand

**DOI:** 10.1002/ece3.70074

**Published:** 2024-07-22

**Authors:** Steph Bennington, Peter W. Dillingham, Scott D. Bourke, Stephen M. Dawson, Elisabeth Slooten, William J. Rayment

**Affiliations:** ^1^ Department of Marine Science University of Otago Dunedin New Zealand; ^2^ Department of Mathematics and Statistics University of Otago Dunedin New Zealand; ^3^ Coastal People Southern Skies Centre of Research Excellence University of Otago Dunedin New Zealand; ^4^ Department of Ecology University of Otago Dunedin New Zealand; ^5^ Department of Zoology University of Otago Dunedin New Zealand

**Keywords:** cetacean, extrapolation, Hector's dolphin, spatial transferability, species distribution modelling

## Abstract

Species distribution models (SDMs) can be used to predict distributions in novel times or space (termed transferability) and fill knowledge gaps for areas that are data poor. In conservation, this can be used to determine the extent of spatial protection required. To understand how well a model transfers spatially, it needs to be independently tested, using data from novel habitats. Here, we test the transferability of SDMs for Hector's dolphin (*Cephalorhynchus hectori*), a culturally important (taonga) and endangered, coastal delphinid, endemic to Aotearoa New Zealand. We collected summer distribution data from three populations from 2021 to 2023. Using Generalised Additive Models, we built presence/absence SDMs for each population and validated the predictive ability of the top models (with TSS and AUC). Then, we tested the transferability of each top model by predicting the distribution of the remaining two populations. SDMs for two populations showed useful performance within their respective areas (Banks Peninsula and Otago), but when used to predict the two areas outside the models' source data, performance declined markedly. SDMs from the third area (Timaru) performed poorly, both for prediction within the source area and when transferred spatially. When data for model building were combined from two areas, results were mixed. Model interpolation was better when presence/absence data from Otago, an area of low density, were combined with data from areas of higher density, but was otherwise poor. The overall poor transferability of SDMs suggests that habitat preferences of Hector's dolphins vary between areas. For these dolphins, population‐specific distribution data should be used for conservation planning. More generally, we demonstrate that a one model fits all approach is not always suitable. When SDMs are used to predict distribution in data‐poor areas an assessment of performance in the new habitat is required, and results should be interpreted with caution.

## Graphical abstract

Species distribution models (SDMs) are commonly used to predict habitat and distribution for species in data poor areas, under the assumption that habitat use is the same across space. We tested the transferability of SDMs for Hector's dolphin, revealing differences in habitat use between populations. These results indicate that to understand habitat use of Hector's dolphin, local data is required for the model building process.

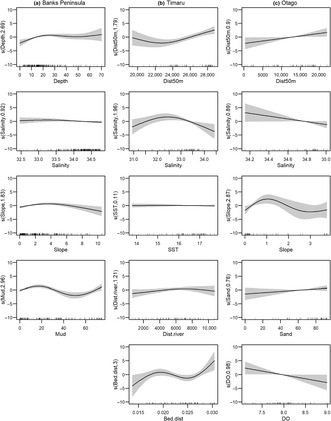

## 1 INTRODUCTION

The distribution of a species reflects its physiological tolerances, as well as the interactions with the environment and wider community (i.e. the ecological niche; Grinnell, 1914; Hirzel & Le Lay, 2008; Hutchinson, 1957; Pulliam, 2000). Beyond allowing scientists to place the species within its ecological niche, understanding a species' distribution and its drivers has broad implications for conservation managers (Hays et al., 2019). Knowledge of species distributions is crucial to planning and implementing conservation actions, including reserve design (Lusseau & Higham, 2004; Silva et al., 2012), identification of high‐priority conservation areas (Ferrier et al., 2002; Kremen et al., 2008), invasive species management (Pheloung et al., 1999; Soberon et al., 2001), and determining suitable areas for translocation (Johnson et al., 2007). Over the past three decades Species Distribution Models (SDMs) have become a widely used tool, primarily for two goals: (1) descriptive modelling, to identify the drivers behind observed distribution patterns (e.g. Bennington et al., 2020; Bräger et al., 2003; Brough et al., 2023; Rizzari et al., 2014; Verhelst et al., 2016), and (2) predictive modelling, for predicting the distribution of a species throughout its range or in unstudied areas or times (e.g. Gantchoff et al., 2022; Olson et al., 2021; Stephenson et al., 2020; Torres et al., 2013, 2015). Descriptive and predictive modelling is often used concurrently, for example, to produce habitat suitability surfaces (Ducci et al., 2015; Laman et al., 2018; Segal et al., 2021). While predictive SDMs are widely used, there are various challenges (Guisan & Thuiller, 2005; Lee‐Yaw et al., 2022; Yates et al., 2018) that can compromise the validity of extrapolating results from one distinct time or space to another, termed *model transferability* (e.g. Thomas & Bovee, 1993).

Model transferability relies on the assumption that the ecological drivers of a species' distribution are similar among locations or through time (Guisan & Thuiller, 2005). There are several reasons why this assumption may not be met: (1) distribution is directly related to the fundamental niche of a species (Soberón & Nakamura, 2009); however, data are usually collected from the realised niche of a population, which may not encompass the species' entire range (Hutchinson, 1957). (2) Environmental variability can result in differences in the importance of the drivers of distribution to specific populations (McAlpine et al., 2008). (3) A population distributed outside the species' fundamental niche (i.e. sink populations, Pulliam, 2000) may persist in environmental conditions that are different. (4) For species that have undergone significant decline, the distribution of remnant populations may reflect the distribution of impacts (e.g. hunting, bycatch), rather than preferred habitat (Channell & Lomolino, 2000). For these reasons, models built using distribution data from one population may not reflect how another uses its available habitat. Furthermore, the transferability of SDMs requires an assumption of distribution equilibrium, i.e. that populations are not rapidly changing their distribution (Guisan & Theurillat, 2000). For taxa of high conservation concern, this assumption is unlikely to be met (Guisan et al., 2013). An alternative approach to transferring SDMs between populations is to develop broad‐scale models, i.e. models constructed using data from the entire fundamental niche (e.g. Stephenson et al., 2020; Torres et al., 2013). However, there are scenarios in which this may not be feasible (e.g. if distribution data are available from only a portion of the species range), and with respect to reason 2 above, broad‐scale models may reflect cumulative distribution patterns. At a fine‐scale, therefore, these models may be unable to accurately discriminate the distribution of a specific population. In these instances, if SDM results are used to predict distributions in novel areas or on finer scales, there should be an obligation to test the predictive performance of the model.

The predictive ability of SDMs is often determined by splitting the source data into separate training and testing datasets (often referred to as cross‐validation, Araújo et al., 2005; Fielding & Bell, 1997). Testing data are withheld from model building, creating an independent dataset that can be used to validate the predictive ability of the model (interpolation). If interpolation is good, the model is assumed to be a good fit in novel areas. Researchers have begun to test this assumption through validating model predictions using real data collected from novel areas or times (i.e. testing the transferability, Duque‐Lazo et al., 2016; Gantchoff et al., 2022; Heinänen et al., 2012; Nguyen et al., 2022; Olson et al., 2021; Petitpierre et al., 2017; Scales et al., 2016; Torres et al., 2015; Vanreusel et al., 2007; Verbruggen et al., 2013). To date, there is little evidence for ubiquitous transferability of SDMs, even when interpolation is considered good. For cetaceans, there are very few published studies on the transferability of SDMs (e.g. Becker et al., 2019; Mannocci et al., 2015, 2017; Monsarrat et al., 2016; Redfern et al., 2017). In one example, Redfern et al. (2017) found that models of the distribution of blue whales (*Balaenoptera musculus*) built with data from only one location were not transferable across space. However, when data from multiple sites were combined, model predictions matched well to the hypothesised distribution.

**FIGURE 1:**
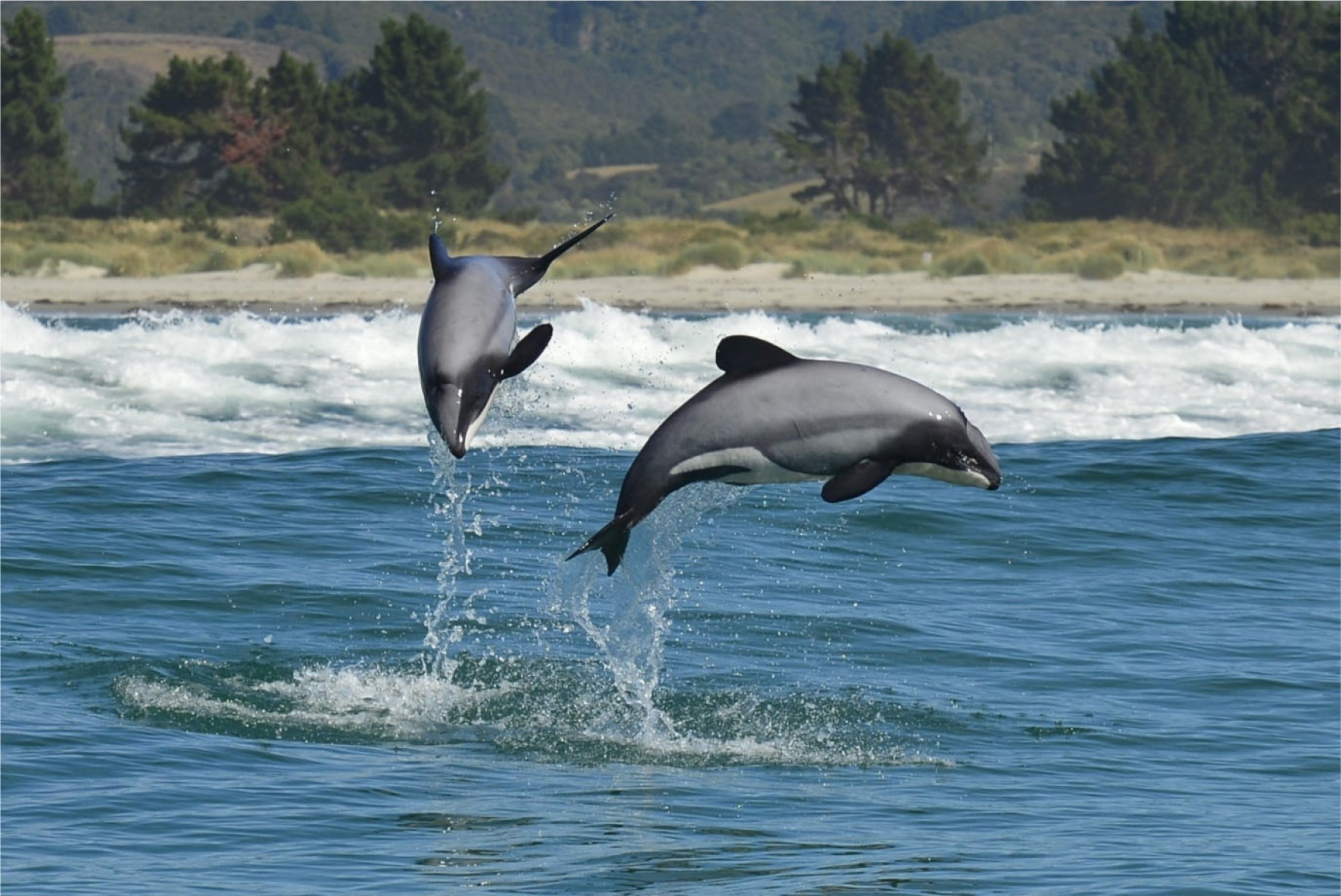
Hector's dolphin (*Cephalorhynchus hectori*) from Otago, New Zealand.

Hector's dolphin (*Cephalorhynchus hectori*, also known as tutmairekurai, pahu, upokohue, Figure 1) is a species of high conservation concern that is endemic to Aotearoa New Zealand (NZ). It is a culturally important (taonga), coastal delphinid, composed of two subspecies: the critically endangered North Island Māui dolphin (*C. hectori maui*, Baker et al., 2002, 2019) and the endangered South Island Hector's dolphin (*C. hectori hectori*, Reeves et al., 2013). Population modelling and studies of mtDNA show that both subspecies have experienced widespread declines since the 1970s (Martien et al., 1999; Pichler et al., 1998; Pichler & Baker, 2000; Slooten & Davies, 2012), mostly due to bycatch in gillnet and trawl fisheries (Dawson, 1991; MacKenzie et al., 2022). Several studies have investigated drivers of distribution for specific populations (Bräger et al., 2003; Brough et al., 2023; Derville et al., 2016; Weir & Sagnol, 2015) or for the entire species (Stephenson et al., 2020; Torres et al., 2013), but none have tested transferability of SDMs. Therefore, it is not clear whether knowledge of habitat preferences of one population can be used to predict distribution for another. This is important, particularly when predictive models are being used to help make conservation decisions, which was the case in the most recent threat management plan for Hector's and Māui dolphin (Department of Conservation, 2020; Roberts et al., 2019).

We tested the spatial transferability of SDMs for Hector's dolphins developed using data collected from three areas along the east coast of the South Island of NZ. Hector's dolphins are most common in inshore waters (e.g. Rayment et al., 2010), and have small alongshore home ranges (approximately 50 km, Rayment et al., 2009). Our study sites were therefore spaced at least 90 km apart so that we were able to test the degree of spatial transferability among distinct geographic areas. Each model used ‘training’ and ‘testing’ data from the source area to validate the predictive performance internally (interpolation), and then was tested for transferability to the remaining two areas. We compared the model performance between the interpolation and transferability results and discuss the implications for Hector's dolphins and other related species.

## 2 METHODS

### 2.1 Study sites

**FIGURE 2:**
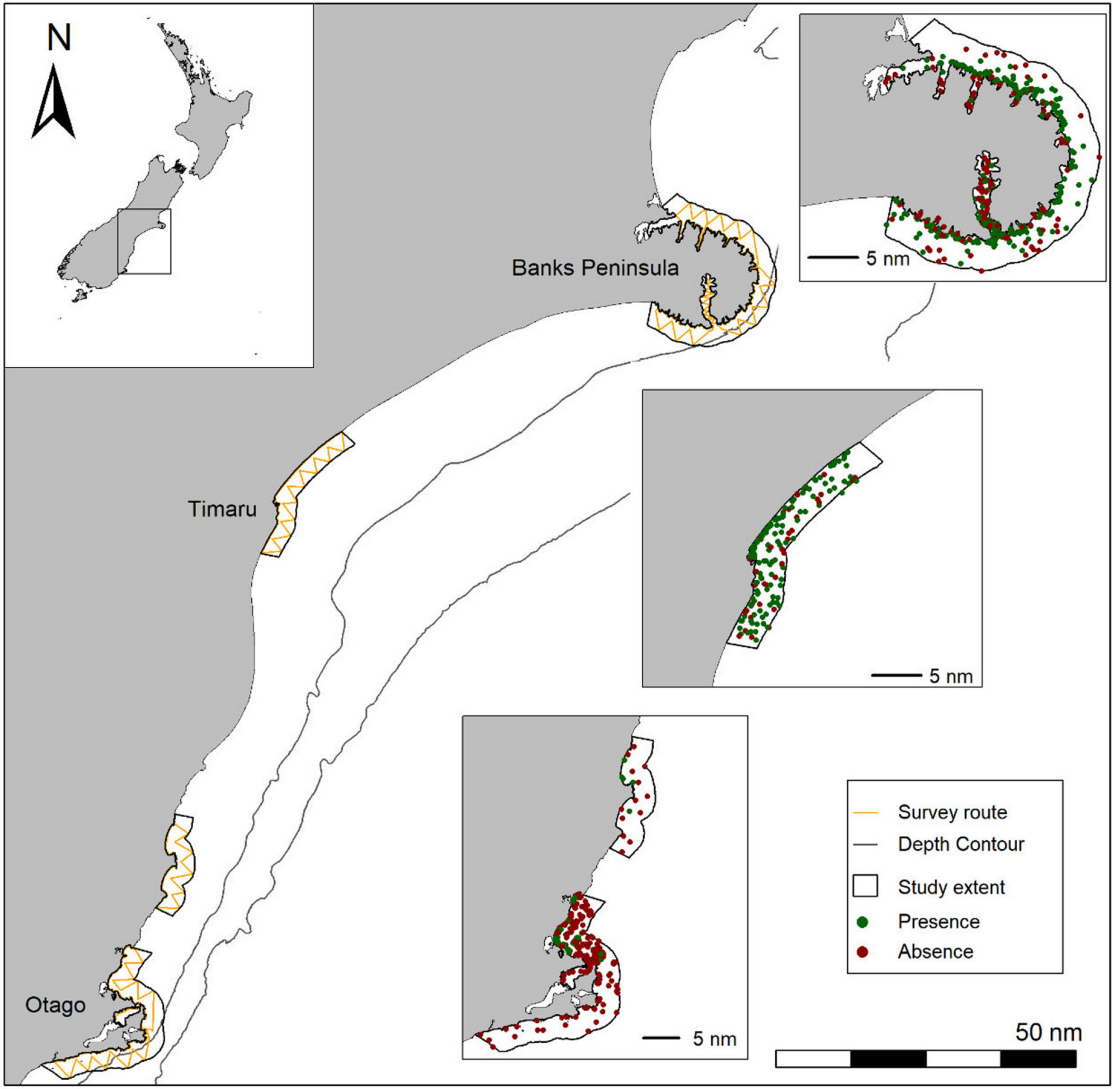
Southeast coast of the South Island of Aotearoa New Zealand with the extent of areas where distribution surveys of Hector's dolphins (*Cephalorhynchus hectori*) were conducted (study areas). Displayed are example survey routes (orange) and the 50 and 100 m isobaths (black). Map insets represent where the coastline is located in New Zealand. Locations where environmental data were collected for Species Distribution Models in the presence (green) and absence (red) of dolphins.

We chose three locations where Hector's dolphin are known to be resident (Figure 2) and which represent a wide range of dolphin density. Banks Peninsula and Timaru are hotspots of dolphin abundance (Dawson et al., 2004; MacKenzie & Clement, 2014), while Otago supports smaller resident sub‐populations (Turek et al., 2013; Williams, 2022). In Banks Peninsula the coastal environment is made up of many bays and two long (>10 km) harbours, with small, steep watersheds. There is a steep decline to the 50 m isobath on the east side of the peninsula, but the south and north sides shelve more gradually (Figure 2). Timaru is characterised by relatively long straight beaches, multiple large watersheds, and a shallow bathymetry which shelves gradually throughout the study area (Figure 2). The Otago area is composed of two peninsulas, a long harbour and a large, semi‐sheltered bay (Blueskin Bay) which hosts smaller bays and long sandy beaches. To the east of Otago Peninsula, depth increases rapidly to the 100 m isobath (Figure 2). The environmental characteristics of Otago were most similar to those of Banks Peninsula, with similar ranges in the covariates used for this study (Appendix S1).

### 2.2 Data collection

We collected distribution data from 8261 km of boat‐based surveys during the austral summers of 2021/2022 and 2022/2023. Surveys were conducted within three nautical miles (nm) of the coastline (Figure 2) using small research vessels (5–6.5 m long, with 70–115 hp outboard engines). While on effort, vessel speed was between 12 and 15 knots and a minimum of two observers continuously scanned the water within 400 m of the vessel looking for dolphins. If dolphins were detected, we slowed the vessel and approached the group to confirm species identification, estimate group size, note behaviour state and collect photographs of marked individuals. Once data from the dolphin group were collected, we returned to the location where individuals were first sighted to record in‐situ environmental data. These location data were used as environmental covariates for ‘presence’ locations in Species Distribution Models (SDMs). During the surveys, if dolphins had not been sighted for a period of 30 min, the vessel was stopped, and a five‐minute stationary survey conducted. Observers scanned a 360° area around the vessel looking for dolphins. If no dolphins were detected, we collected environmental data from that location to be used as an ‘absence’ location. If dolphins were detected during the stationary survey, the absence data were disregarded, and environmental data were collected from the location of the new group.

Sea surface temperature and depth at all presence and absence locations were measured via the onboard echosounder. Depth profiles of temperature (°C), salinity (PSU), dissolved oxygen (mg L^−1^) and fluorescence (μg L^−1^) were recorded using a Sea‐Bird CTD (SBE‐192267, Electronics Inc., Bellevue, Washington) or RBR concerto (Ruskin, RBR Ltd. Ottawa, Ontario, Canada). Turbidity data were collected by lowering a Secchi disk (30 cm diameter) until it was no longer visible from the surface. The vessel's on‐effort track was recorded every 30 s, using the inbuilt Global Positioning System of a Samsung Galaxy tablet. Sighting and environmental data were collected within a Cybertracker (© CyberTracker Conservation, 2021) custom‐built data collection application, running on the tablet. All fieldwork followed the regulations outlined in the NZ Marine Mammal Protection Act (1978) and Marine Mammal Protection Regulations (1992).

### 2.3 Species distribution modelling

We used generalised additive models (GAMs, Wood, 2017a) to investigate how well SDMs transferred among populations of Hector's dolphin at Banks Peninsula, Timaru and Otago. GAMs provide a non‐parametric approach for modelling species distributions (Hastie & Tibshirani, 1986; Wood, 2017a), are able to identify the potential underlying drivers of distribution (Bell & Schlaepfer, 2016; Derville et al., 2018), and have been a useful tool in understanding the distribution of cetaceans (e.g. Bennington et al., 2020; Redfern et al., 2017; Torres et al., 2008). Furthermore, in terms of transferability, their performance is similar when compared to other modelling approaches (Derville et al., 2018; Heikkinen et al., 2012).

GAMs were built in R v.4.3.1 (R Core Team, 2023) within the R Studio v.2023.12.0.369 (Posit team, 2023) environment using the package ‘mgcv’ (Wood, 2017b). The binary response (presence or absence of dolphins) was related to a suite of continuous environmental covariates, using a ‘logit’ link, and thin‐plate regression splines. We limited covariates to a maximum of four degrees of freedom to reduce the risk of overfitting (e.g. Rayment et al., 2015).

### 2.4 Response variable

Presence and absence locations of Hector's dolphin were collected in situ during surveys as described above. To reduce the risk of spatial autocorrelation, if presence locations were within 400 m of the previous presence (a distance within which Hector's dolphins typically have a positive response to the vessel, Dawson et al., 2004), only the initial presence was retained. If dolphins were detected within 400 m of an absence location, the absence was disregarded, and the presence location was used instead. Dolphins are highly mobile animals; although we attempted to ensure there were no dolphins present at absence locations during the time of sampling, individuals are able to use the area prior to and after sampling (Fernandez et al., 2022). For this reason, absence locations are considered pseudo‐absences in this analysis.

### 2.5 Environmental covariates

In addition to the temporally dynamic covariates gathered in situ during surveys, we associated temporally static covariates with the locations of dolphin presence and absence points. These included the slope and aspect of the sea floor (°), substrate type (% composition of mud, sand, gravel), benthic sediment disturbance (Bed.dist, m s^−1^) and distance to nearest river mouth (Dist. river, m), coast (Dist.coast, m), 50 m isobath (Dist50m, m) and 100 m isobath (Dist100m, m). Substrate covariates were generated from raster data (200 × 200 m resolution) calculated as part of a seabed mapping project (Bostock et al., 2019), and obtained from the National Institute of Water and Atmospheric Research (NIWA). We also included the depth averaged tidal current speed (tidal, m s^−1^, 200 × 200 m resolution) calculated from a tidal model for NZ (Walters et al., 2001). The covariates used in this study have been included for species distribution models of NZ dolphins in the past (Bräger et al., 2003; Brough et al., 2023; Derville et al., 2016) or, in the case of dissolved oxygen, can act as a proxy for other environmental variables (e.g. indicating areas of high primary productivity, and potentially good foraging habitat).

All covariates were checked for concurvity, a post‐hoc check which allows for non‐linear dependencies between covariates in the model to be described (Amodio et al., 2014). After fitting the full suite of covariates in the model, covariates with concurvity higher than 0.3 were not included together in further models (e.g. He et al., 2006). For each pair of correlated covariates, the one with the highest deviance explained in univariate models was retained for model selection (e.g. Bennington et al., 2020).

### 2.6 Model building

The ratio of absence to presence locations is an important consideration when constructing binomial SDMs (Barbet‐Massin et al., 2012; Fernandez et al., 2022). As we collected absence data in‐situ, the ratio of absences to presences was controlled by encounter rates at each site. For Banks Peninsula the absence to presence ratio occurred at approximately 1:2, in Timaru approximately 1:3, and in Otago approximately 5:1. To ensure that models were comparable at each site, we randomly (without replacement) down‐sampled the presence data in Banks Peninsula and Timaru and the absence data in Otago, so that models were built with an equal number of absences and presences (as recommended by Barbet‐Massin et al., 2012). To ensure that transferability results were not the product of the subset of data used, we repeated the randomised down‐sampling for all three sites. Ten datasets were generated for Banks Peninsula, using all available absence data and 10 random subsets of the presence data. As Timaru and Otago both had approximately half the amount of data collected in comparison to Banks Peninsula, we generated half the number of datasets (five each).

Non‐concurved environmental covariates were fit into a GAM object for each randomly generated dataset. Models then underwent a model selection procedure using the double‐penalty approach (Marra & Wood, 2011; Wood, 2017a), to determine the most influential covariates. This method minimises the Unbiased Risk Estimator (UBRE) score by applying an additional shrinkage term to each covariate allowing smooth terms to be shrunk to zero, thus having no influence on the response (Wood, 2017a). Similar to Akaike's Information Criterion (Akaike, 1973), the double‐penalty approach maximises fit while penalising complexity, and the model with the lowest UBRE value is considered the top model. We applied the double‐penalty approach with the default settings in the ‘mgcv’ package in R.

### 2.7 Model validation

We used multi‐fold model validation (also referred to as k‐fold cross‐validation, e.g. Hastie et al., 2001) to test the predictive ability of top models, using data collected within the same area (i.e. model interpolation). Datasets were partitioned into three folds by random sampling without replacement. Two of the folds were combined and used to build and select the top model (i.e. the ‘training’ data), and the third fold was used as an independent dataset which was withheld from the model building process (i.e. ‘testing’ data). The probability of Hector's dolphin presence was predicted for each location contained within the testing data using the top model and compared to the true response (presence or absence). The True Skill Statistic (TSS) and Area Under the receiver operator Characteristic curve (AUC) were generated and used to assess interpolation (Allouche et al., 2006; Lobo et al., 2008). TSS values range between negative one and positive one; a model with perfect sensitivity (the proportion of correctly predicted presences) and specificity (the proportion of correctly predicted absences) will have a TSS of one, and models that perform the same as randomly assigning values have a TSS of zero. We used a probability threshold of 0.5, as recommended by Lobo et al. (2008) for instances where there is an equal ratio of presence and absences in the training data (e.g. Rayment et al., 2015). AUC is a threshold independent measure of model accuracy, which takes a value between 0.5 and one. Generally, an AUC score above 0.7 is considered a *useful* model (sensu Hosmer Jr. et al., 2013). Lobo et al. (2008) recommend reporting sensitivity and specificity alongside AUC so that the importance of omission (proportion of incorrectly predicted absences) and commission (proportion of incorrectly predicted presences) errors can be assessed. Here, we report the sensitivity and specificity as well as TSS (e.g. Rayment et al., 2015).

To examine autocorrelation, we generated a variogram of residuals for each top model (e.g. Wood, 2017a) using the variog function from the ‘geoR’ package (Ribeiro et al., 2022). The semivariance generally did not increase with distance for the best models in any of the three areas, suggesting that spatial autocorrelation was not an issue at the scale of our three study areas.

### 2.8 Model transferability

We tested the transferability of SDMs for Hector's dolphin by using models created for each site to predict the distribution at the two sites outside of the model's source area (e.g. Gantchoff et al., 2022; Olson et al., 2021; Redfern et al., 2017). For example, models built with data sourced from Banks Peninsula were used to predict the distribution of dolphins in both Otago and Timaru. This was repeated for each of the top models in the three‐fold validation and for each dataset. We generated TSS and AUC validation statistics for transferability and compared these to the interpolation values.

To understand how model transferability was influenced by the source data, we built ‘regional’ models using data from two of the three locations and tested the spatial transferability to the remaining area. We followed the same procedure as described above. The regional data were first tested for concurvity, then randomly downsampled into 10 unique datasets for each region: Banks Peninsula and Timaru (region 1), Banks Peninsula and Otago (region 2), and Timaru and Otago (region 3). Datasets were partitioned into three‐folds, by randomly sampling the data (without replacement). Model interpolation performance was first assessed by generating sensitivity, specificity, TSS and AUC values. Then spatial transferability was assessed with TSS and AUC by using the model to predict the distribution of Hector's dolphin in the remaining area.

## 3 RESULTS

**FIGURE 3:**
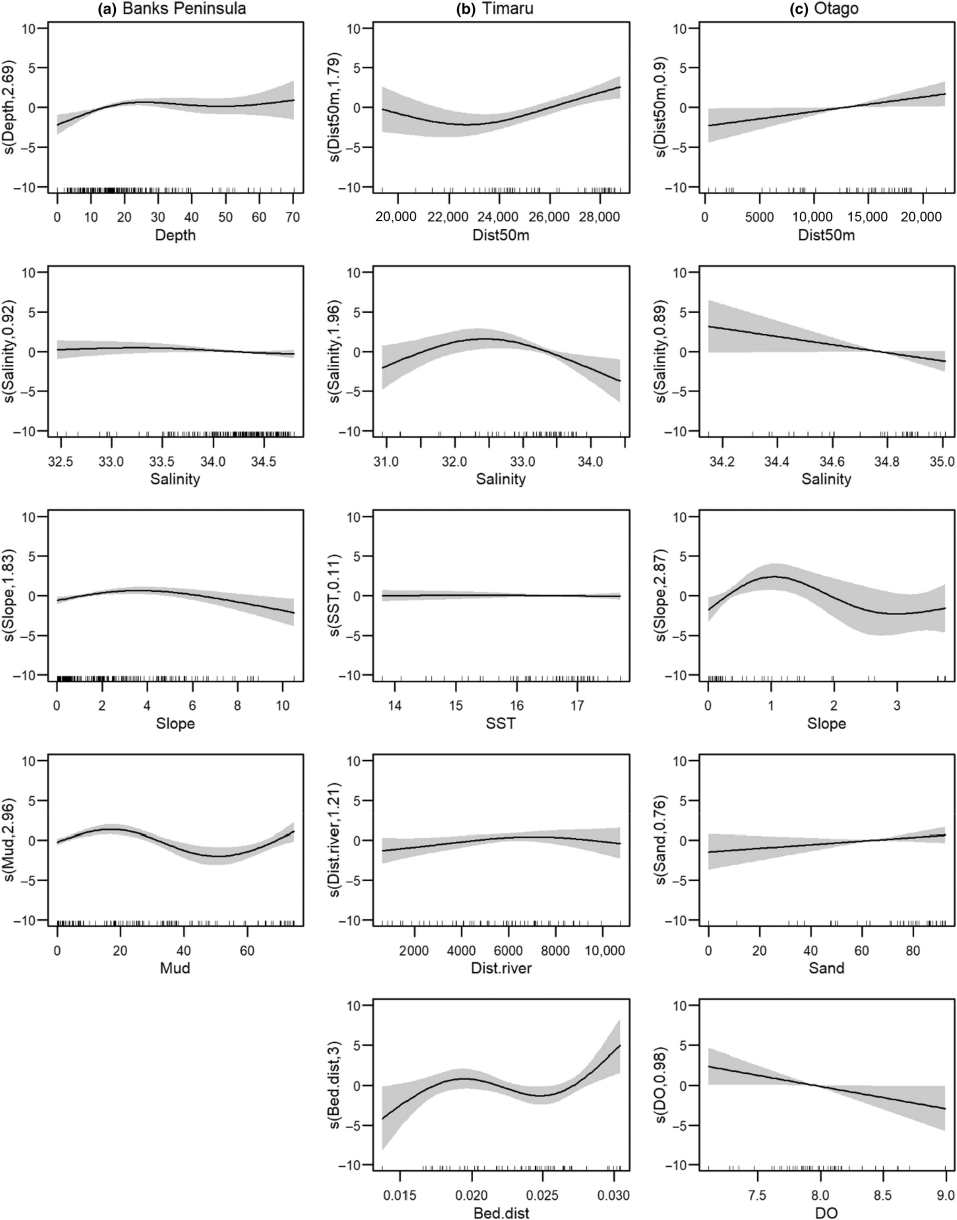
Effect of environmental covariates from logistic generalised additive models on the presence of Hector's dolphin (*Cephalorhynchus hectori*) at Banks Peninsula, Timaru, and Otago. Displayed are example relationships from the best performing models (combination of the highest interpolation and deviance explained). The 95% confidence interval of the response is represented by the shaded area. The *y*‐axis shows the centred smooth function of each variable, with the estimated degrees of freedom. RUG plots, along the *x*‐axis of each plot, show the distribution of data across the range of each variable. All covariates are displayed across the same scale and beside similar covariates from the other sites, for comparative purposes. Variables include: Depth (m), Slope (°), percent substrate composition of sand (Sand, %) or mud (Mud, %) and the disturbance (Bed.dist, m s^−1^, Bostock et al., 2019), of the seafloor, as well as the sea surface temperature (SST, °C), dissolved oxygen (DO, mg L^−1^), Salinity (PSU) and the distance to the nearest river (Dist. river, m) or the 50 m isobath (Dist50m, m).

Over the 2021/22 and 2022/23 austral summers, we collected environmental covariates from 537 groups of Hector's dolphins, and at 441 locations where dolphins were absent. In total, 440 presence and 399 absence locations were of suitable quality for model building (i.e. were further than 400 m apart, and had a complete suite of covariates, Figure 2; Table 1). At each site, different suites of covariates were retained in the best performing models (Table 2). In Banks Peninsula, dolphins showed general preferences for areas approximately 20–30 m deep, slight slopes (approximately 4°), and with a substrate composed of approximately 20% mud (Figure 3a). The relationship with salinity was generally weak and varied between top models. Dolphins in Timaru were more likely to be found in areas further from the 50 m isobath, with low salinities (approximately 32.5PSU), increasing distance from river mouths, and with higher seabed disturbance (up to 0.3 m s^−1^, Figure 3b). The relationship with SST was generally weak and varied between models. Otago models indicated that dolphins were most likely to be found further from the 50 m isobath, and in areas with lower salinities (approximately 34.2PSU), slight slopes (1°), where the substrate had a high proportion of sand, and lower dissolved oxygen concentrations (Figure 3c). When similar covariates were retained between areas (e.g. dissolved oxygen in Banks Peninsula and Otago, or salinity and distance to the 50 m isobath in Otago and Timaru) the relationships or ranges of covariates were seldom consistent (Figure 3).

**TABLE 1:** Summary of survey effort for Hector's dolphins (*Cephalorhynchus hectori*) across sites on the southeast coast of the South Island, Aotearoa New Zealand: Banks Peninsula (BP), Timaru (TIM) and Otago (OTA), in the austral summers of 2021/22 and 2022/23.

Location	2021/22			2022/23			SDM response	
	Surveys	Distance surveyed (km)	Dolphin groups	Surveys	Distance surveyed (km)	Dolphin groups	Presence	Absence
BP	33	2405	245	28	2118	164	270	163
TIM	10	554	61	9	574	97	129	39
OTA	19	1680	28	11	930	29	41	197
Total	62	4639	334	48	3622	290	440	399

*Note*: SDM response shows the number of presence and absence locations that were suitable for use in species distribution models.

**TABLE 2:** Summary of the mean logistic generalised additive model interpolation (Interp) and spatial transferability (Transfer) results from three locations along the southeast coast of the South Island: Banks Peninsula (BP), Timaru (TIM), and Otago (OTA), and three regional models built with data from: BP and TIM (R1), BP and OTA (R2), and OTA and TIM (R3).

Site		Covariates	Adj. *R* ^2^	Dev. Exp.	Sen	Spe	TSS (95% CI)		AUC (95% CI)	
							Interp	Transfer	Interp	Transfer
Single site models	BP	Depth, Slope, Mud, DO, Sal	.17	0.16	0.67	0.63	0.30 (0.10, 0.50)	−0.11 (−0.26, 0.05)	0.71 (0.60, 0.82)	0.60 (0.55, 0.64)
	TIM	Sal, Bed.dist, Dist50m, SST, Dist.river	.26	0.29	0.55	0.55	0.07 (−0.36, 0.50)	0.00 (−0.18, 0.19)	0.58 (0.40, 0.76)	0.61 (0.46, 0.77)
	OTA	Slope, Sal, DO, Dist50m, SST, Sand	.30	0.30	0.69	0.64	0.32 (0.02, 0.62)	−0.04 (−0.08, 0.00)	0.73 (0.48, 0.99)	0.56 (0.50, 0.62)
Regional modes	R1	Slope, Gravel, Bed.dist, SST, DO, Dist100m	.15	0.14	0.65	0.60	0.25 (0.10, 0.41)	−0.03 (−0.11, 0.05)	0.66 (0.55, 0.77)	0.55 (0.42, 0.69)
	R2	Depth, Slope, Aspect, Gravel, SST, DO, Dist100m	.32	0.27	0.77	0.69	0.46 (0.28, 0.64)	0.04 (−0.06, 0.14)	0.79 (0.71, 0.87)	0.74 (0.70, 0.78)
	R3	Slope, Mud, SST, DO, Dist50m	.43	0.36	0.83	0.80	0.85 (0.47, 0.77)	0.30 (0.07, 0.54)	0.85 (0.75, 0.96)	0.79 (0.76, 0.82)

*Note*: All model results produced across the three‐fold validation and multiple datasets were averaged for each location. Included are the covariates of the species distribution models: Depth (m), Slope (% change), benthic sediment disturbance (Bed.dist, m s^−1^, Bostock et al., 2019), distance to the 50 m depth contour (Dist.50 m, m), distance to the nearest watershed outlet (Dist. river, m), percent composition of mud, sand or gravel in the substrate (Mud/Sand/Gravel), the concentration of dissolved oxygen (mg L^−1^), surface salinity (Sal, PSU), and sea surface temperature (SST, °C). Adjusted *R* ^2^ (Adj. *R* ^2^), proportion of deviance in the response that is explained by the model (Dev. explained), the sensitivity (Sen), the specificity (Spe), true skills statistic (TSS) and the area under the receiver operator curve (AUC) with the associated 95% confidence interval (CI) in brackets.

On average, models from Otago performed better than both Banks Peninsula and Timaru. The mean deviance explained and adjusted *R* ^2^ of the Otago models were the highest of all three sites, while Banks Peninsula had the lowest (Table 2). Interpolation scores were similar between Otago and Banks Peninsula, generally indicating useful models (TSS > 0, AUC > 0.7), with lower variability in Banks Peninsula. For Timaru models, interpolation scores were poor, showing the lowest average TSS and AUC, indicating models were predicting no better than random.

**FIGURE 4:**
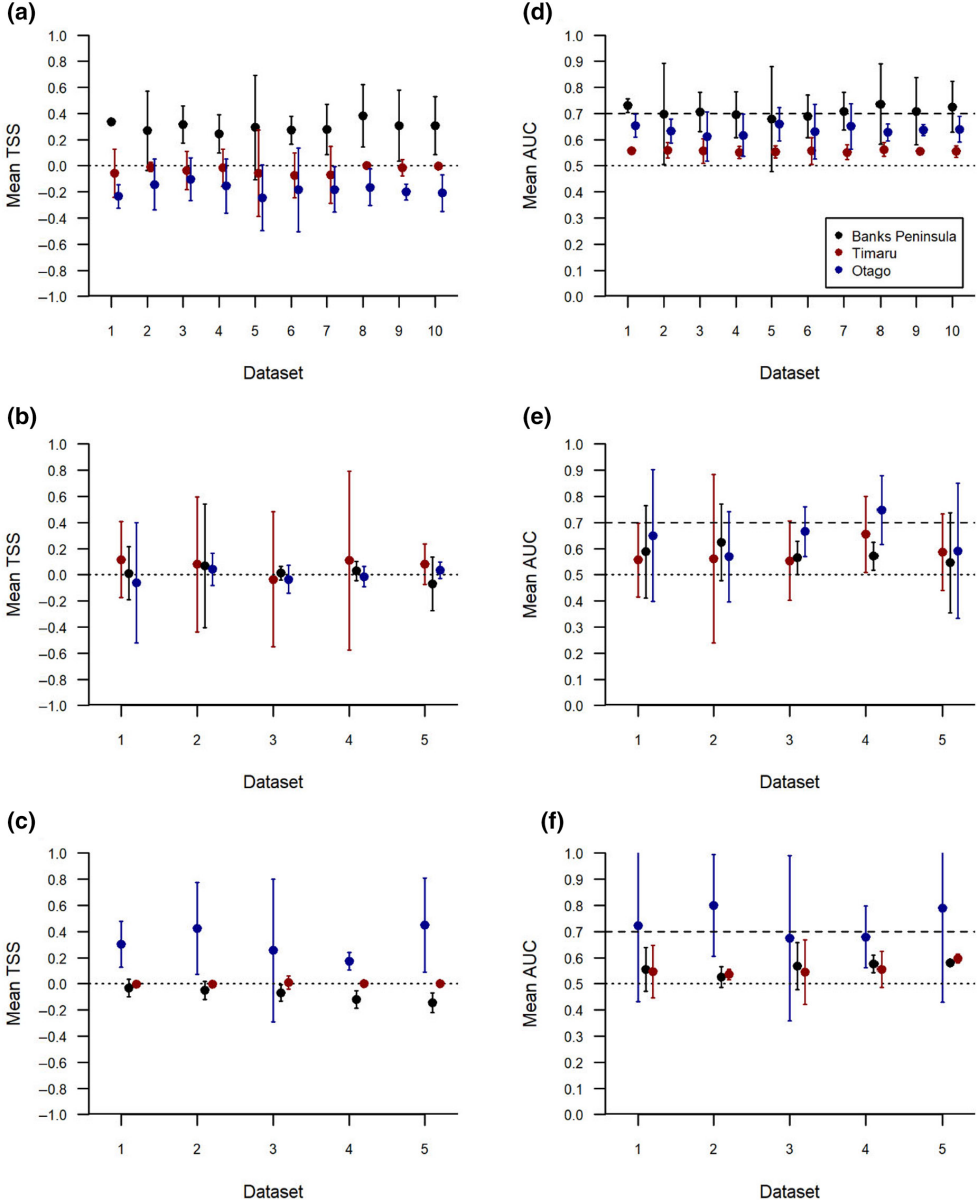
Comparison of the interpolation and transferability of species distribution models for Hector's dolphins (*Cephalorhynchus hectori*) with data sourced from three locations along the southeast coast of the South Island, New Zealand: Banks Peninsula (a and d), Timaru (b and e), and Otago (c and f). The *x*‐axis shows the randomly selected subset of data (Dataset) used for model building. Displayed are the mean TSS and AUC values, with the associated 95% confidence intervals of three‐fold validated logistic generalised additive models. Model interpolation results are given when the data source matches the colour code, and transferability results when the data source does not match the colour code. For TSS, the dotted line, and for AUC between the dotted and dashed lines, indicates the value below which the model is no better at predicting the distribution than random.

**FIGURE 5:**
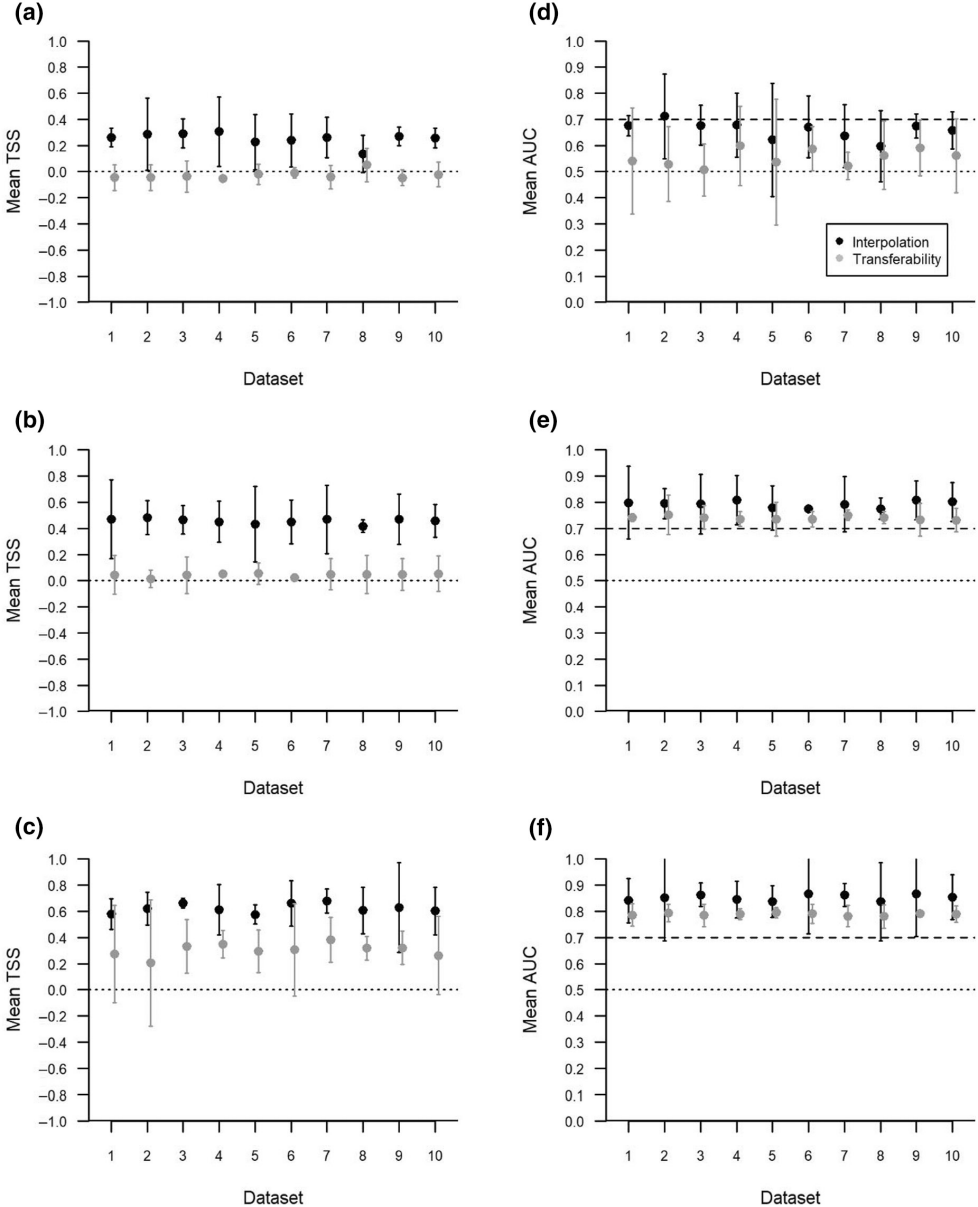
Comparison of the interpolation and transferability of species distribution models for Hector's dolphins (*Cephalorhynchus hectori*) for regional models from the southeast coast of the South Island, New Zealand. Models were built using data sourced from two locations and transferred to the third: Region 1 = Banks Peninsula and Timaru (a and d), region 2 = Banks Peninsula and Otago (b and e), and region 3 = Otago and Timaru (c and f). The *x*‐axis shows the randomly selected subset of data (Dataset) used for model building. Displayed are the mean TSS and AUC values, with the associated 95% confidence intervals of three‐fold validated logistic generalised additive models. Model interpolation results are given when the data source matches the colour code, and transferability results when the data source does not match the colour code. For TSS, the dotted line, and for AUC between the dotted and dashed lines, indicates the value below which the model is no better at predicting the distribution than random.

When transferred to a new area, none of the models performed any better than random (Figures 4 and 5). Mean TSS values for transferability were less than or equal to zero, regardless of how well the model was able to interpolate within its source area (Table 2, Figure 4). Generally, AUC values were lower when the model was transferred to a different area (Figure 4). The exception to this is Timaru, where top models were never capable of predicting distribution better than random for either interpolation or transferability.

Models built with data sourced from two areas generally had stronger interpolation than those built from only one area; however, performance declined when transferred to a new area (Figure 5). This trend was strongest for TSS, where transferability resulted in performance no better than random for regions one and two (Figure 5a,b). Although region three transferred better, TSS had wide confidence intervals which regularly crossed zero (Figure 5c). The trend for AUC was mixed, although there was a decline in performance in all transferred areas, both region two and three models were able to predict the new area better than random (Figure 5e,f).

## 4 DISCUSSION

The predictive ability of Species Distribution Models (SDMs) is regularly cited as a tool to aid in the development of spatial protection (Guisan et al., 2013). It is particularly useful in allowing distributions to be predicted in areas that are data poor. Formal tests of transferability have become more common over the past two decades (Barnes et al., 2014; De Albuquerque et al., 2023; Duque‐Lazo et al., 2016; Gantchoff et al., 2022; Heinänen et al., 2012; Olson et al., 2021; Torres et al., 2015; Vanreusel et al., 2007; Verbruggen et al., 2013; Wogan, 2016). To date, however, results from transferability tests have been varied. Therefore, it is important to independently test how well SDM predictions perform outside of the area where data were collected, particularly when they are used to determine the placement of spatial protection. Two areas in our study (Otago and Banks Peninsula) produced models that would be considered useful for predicting the distribution of Hector's dolphins within the original study site. However, when transferred to novel areas, no model could be considered to perform better than randomly assigning presences and absences. The third area in our study (Timaru) produced models with poor interpolation and transferability statistics.

Poor transferability of SDMs could have several explanations. For this study, potential explanations include: (1) differences in the environmental characteristics of study sites, (2) different habitat preferences among populations, and (3) important covariates missing from the models. Model transferability is most successful among similar habitats (Yates et al., 2018), but this limits the application of model predictions for species that inhabit a wide range of environmental characteristics (i.e. generalist species, Wogan, 2016). When we tested the transferability between even the most similar habitats (e.g. Banks Peninsula and Otago, Appendix S1), the best models were unable to accurately predict the new area. We show that ubiquitous spatial transfer of SDMs for Hector's dolphins, which are found throughout the South Island inhabiting a range of different conditions (Bejder & Dawson, 2001; Derville et al., 2016; Ferreira & Roberts, 2003; Harvey et al., 2022; MacKenzie & Clement, 2014, 2016, 2019; Turek et al., 2013), is not guaranteed. It has been shown that using SDMs with data sourced from multiple populations transfer better to novel spatial areas (e.g. Canadian lynx, *Lynx canadensis*, Olson et al., 2021; blue whales, *Balaenoptera musculus*, Redfern et al., 2017). Although there is some evidence this may be the case for Hector's dolphin, we note this depends on the validation statistic used (e.g. AUC showed better transferability then TSS) and only when data from Otago were included in model building. Otago had low densities of dolphins (fewer occurrence locations and lower estimated abundances, e.g. MacKenzie & Clement, 2014), so combining data with that of Banks Peninsula or Timaru results in the majority of absence data sourced from one area and the majority of presence data sourced from the other. In general, regional transfer produced mixed results with indications of poor transferability. For TSS, the declines resulted in models that were generally unable to predict any better than random in the new area.

We show that top models built with data from different areas generally retained different explanatory variables. Furthermore, even when the same covariates were retained in the top models, their relationships with dolphin presence varied. Overall, our results provide interesting insight into differing habitat use of dolphins living at Banks Peninsula, Timaru and Otago. Bräger et al. (2003) made a similar conclusion, detecting significant differences in habitat selection of dolphins among six study areas. Of the covariates that were included in our models, most have been previously used in other SDMs for Hector's or Māui dolphin. Previous models have used a range of approaches, with different covariates, and algorithms. When models have included new variables (e.g. prey, Brough et al., 2023), which dolphin presence has a strong response to, other variables may approach irrelevance. This can make direct comparisons of modelled relationships challenging. Despite this paradox, with some covariates, our models showed similar patterns to previously described relationships. For example, depth is a consistently important variable in SDMs for Hector's and Māui dolphin, generally indicating a preference for shallower water (less than 50 m, e.g. Bräger et al., 2003; Brough et al., 2023; Derville et al., 2016; Stephenson et al., 2020). We show similar trends, with depth, or covariates related to depth (e.g. distance to the 50 m isobath), indicating a preference for shallower habitats. Another example is where we observed higher probability of occurrence at Banks Peninsula at both low and high proportions of mud in the substrate. This is essentially the same habitat preference demonstrated by Brough et al. (2023) for distribution with respect to sand. Our models, however, also showed distinct differences from previous studies. For example, when SST was retained, we did not observe an increase in dolphin presence with temperatures, unlike both Brough et al. (2023) and Derville et al. (2016). Further, when sand was retained in Otago, dolphins preferred areas of higher sand composition (increasing from 60% to 100%). The differences in SDM results in our study, and when put in context of the literature, suggest that Hector's dolphin habitat preferences vary among areas. This variation likely contributes to the poor model transferability observed in our study.

At best, our SDMs explained just over half of the variation in the data, and on average this was closer to a quarter. Although this is similar to previous SDMs for Hector's and Māui dolphin (e.g. 47% in Brough et al., 2023; 31.3% in Derville et al., 2016; 26.1% in Roberts et al., 2019), it indicates that important covariates may be missing. One such variable is prey, which may be an important covariate for predicting distributions of cetaceans (Pendleton et al., 2020; Stephenson et al., 2023) and has been shown to improve performance of SDMs for dolphins (Bennington et al., 2020; Brough et al., 2023). Biotic variables, such as the presence and absence of prey and predators, are more likely than abiotic predictors to directly affect distributional patterns (e.g. Torres et al., 2008). However, the distribution and relative abundance of fish is dynamic and difficult to quantify (Chrysafi & Kuparinen, 2016). Collection of these data, and linking them to dolphin distribution at a scale that is temporally and spatially relevant, can be equally challenging (e.g. Torres et al., 2008). Furthermore, dolphins are generally opportunistic feeders, and the foraging ecology of Hector's dolphins has been shown to vary between habitats (e.g. Miller et al., 2013; Ogilvy et al., 2023). Incorporating prey into models is therefore likely to result in improved interpolation, however, we believe it is unlikely that model transferability would improve, at least at small spatial scales. How inclusion of biotic variables affects model transferability is untested for Hector's dolphins and would be an interesting area for further research.

The interpolation ability of top models was highly variable, both within and among sites. This could be explained by differing model inputs or greater contrast in covariates between presence and absence locations. As we used consistent methodologies for data collection and model building among all areas investigated, both explanations likely stem from different characteristics of the populations or habitats. For example, the distribution of dolphin presence locations showed greater aggregation in Otago and Banks Peninsula, whereas in Timaru the distribution was more random across the survey area. Fiedler et al. (2023) showed a similar pattern of poor model performance for the more randomly distributed Cuvier's beaked whale (*Ziphius cavirostris*) and sperm whale (*Physeter macrocephalus*), in comparison to the more aggregated distribution of the Baird's beaked whale (*Berardius bairdii*). Alternatively, models may be influenced by dolphin density, which was much lower in Otago, compared to Timaru and Banks Peninsula. Variable density resulted in different sample sizes used in model building, which can influence model performance (e.g. Hallman & Robinson, 2020). Values from one location in Otago or Timaru would have a stronger influence on the modelled relationship than in Banks Peninsula.

Density could also provide an ecological explanation for the differences in interpolation performance among study sites. As density increases, intraspecific competition for space could become more important. This is known as the ‘buffer effect’ (as described by Brown, 1969); when a population has fewer individuals, all are able to occupy high‐quality habitat. Such was the case for bank (*Myodes glareolus*) and field voles (*Microtus agrestis*), where low densities resulted in individuals becoming restricted to core habitat (Sundell et al., 2012). If this is occurring for Hector's dolphin, better contrast in covariates between presences and absences would exist in Otago, resulting in improved ability to discriminate between presence and absence locations by the SDMs. As the Banks Peninsula models had similar performance to Otago, it is unlikely that density dependencies were the only driver of interpolation performance. Further, all three populations likely exist below their original population size (e.g. Slooten & Davies, 2012), therefore contemporary populations are unlikely to have approached carrying capacity so competitive interactions are likely to be minimal. Hector's dolphins are capable of using a wide range of environmental conditions, in areas of low density we are less likely to observe them overall, but particularly in areas they use less. This could create pseudo‐contrast in the model, where it appears to perform better with less information. Greater contrast is also likely affected by the availability and structural complexity of habitat (e.g. Ferrari et al., 2018). In Timaru, covariates existed in a much narrower range (Appendix S1); therefore the environmental characteristics of presences and absences would be similar, lowering the ability of models to discriminate. If sampling occurred across wider environmental gradients, it is likely that model interpolation would improve. Expanding the study extent to the 50 m isobath in Timaru may have enabled greater sampling of absence locations, as occurred in Banks Peninsula and Otago, providing greater environmental ranges. However, we note that better interpolation is not necessarily linked to improved transferability (as shown in the Banks Peninsula and Otago models).

### 4.1 Conservation implications

Species distribution models which accurately predict distributions in novel areas can be valuable for conservation management (Guisan et al., 2013). Collecting distribution data throughout the entire range of a species is often challenging and expensive. Even when that investment has been made, small populations can be missed, and some may be inaccessible. For data poor populations or areas, accurate models can help to identify critical habitat (e.g. Heinrichs et al., 2010; Purdon et al., 2020), the occurrence of rare species or populations outside of surveyed areas (e.g. McCune, 2016; Verutes et al., 2021), and areas which were previously inhabited, or may be suitable for future use (e.g. Gantchoff et al., 2022), and therefore suitable for translocations (e.g. Johnson et al., 2007; Nneji et al., 2020). When spatial transferability of SDMs has been tested, however, success has been inconsistent (Yates et al., 2018). This creates a need to assess transferability on a case‐by‐case basis, especially when spatial predictions are used in conservation planning.

**FIGURE 6:**
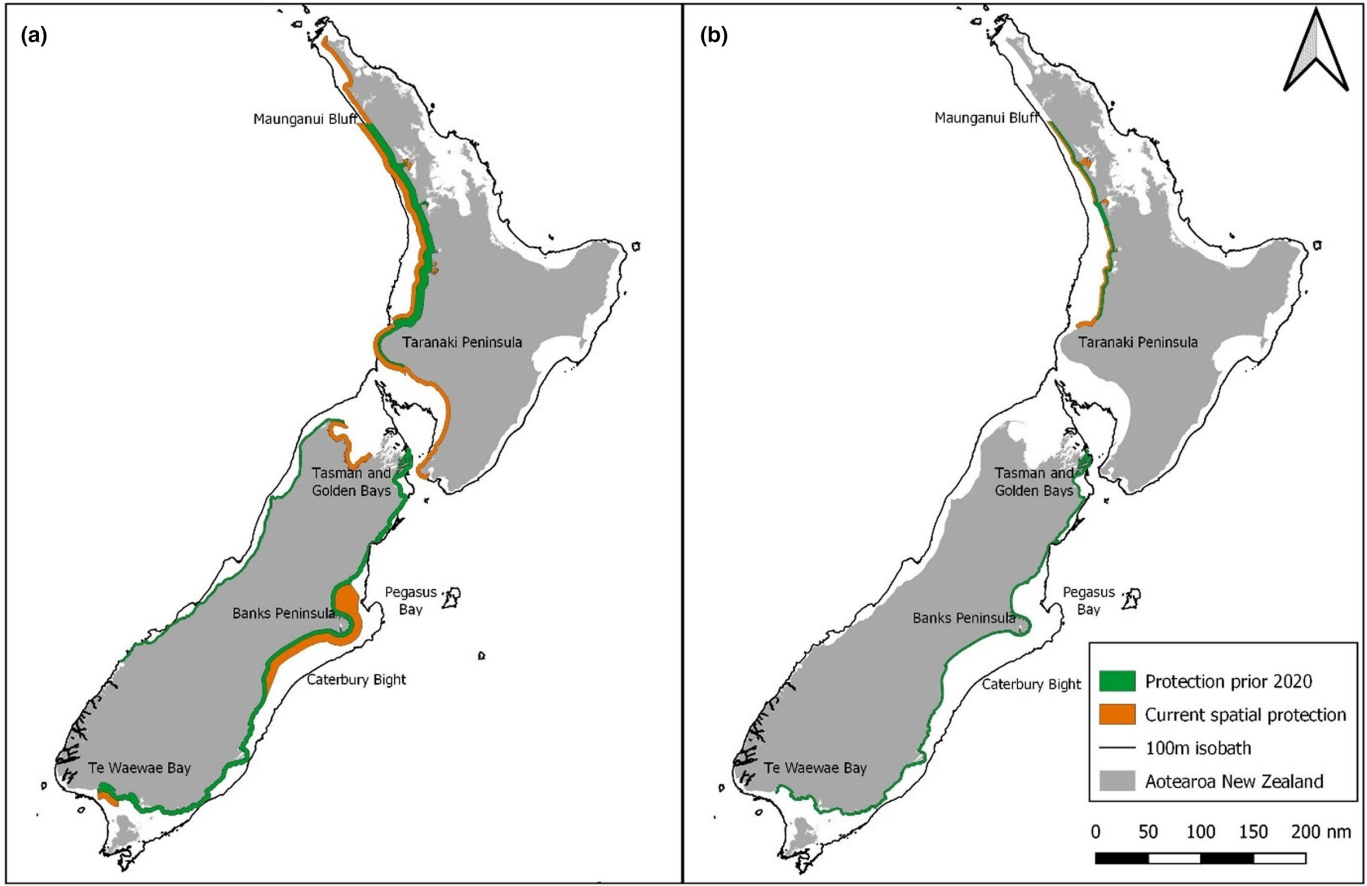
Summary of the pre‐2020 (green) and post‐2020 (orange) spatial restrictions of (a) commercial gillnet, and (b) commercial trawl fisheries around Aotearoa New Zealand. For context, we display the 100 m isobath, which is thought to be the general habitat limit for Hector's and Māui dolphin.

In 2020, the NZ government introduced major revisions to the spatial management of commercial fisheries in an attempt to better protect Hector's and Māui dolphin (Department of Conservation, 2020, see Figure 6). The revisions were justified, in part, by a spatially explicit fisheries risk assessment (SEFRA) that included an SDM (Roberts et al., 2019). Spatially explicit risk assessments have been used in a range of different systems, and provide a method to estimate the impacts of threats across a landscape for a species (Duggan et al., 2015; Heinrichs et al., 2010; Rouget, 2002). For example, Hickcox et al. (2023) used SDMs to understand the overlap in distribution of endangered yellow‐eyed penguins (*Megadyptes antipodes*) with commercial fisheries, and proposed marine protected areas (MPAs) off the east coast of the South Island of NZ. They demonstrated that the proposed MPAs would do little to protect penguin habitat. For the Hector's and Māui dolphin SEFRA, the SDM was built using occurrence data collected during intensive aerial surveys conducted throughout the South Island (MacKenzie & Clement, 2014, 2016, 2019). However, in some regions with low dolphin densities (e.g. Otago; Turek et al., 2013), no sightings were made. Furthermore, no survey effort was conducted in Māui dolphin habitat off the North Island. Despite the lack of occurrence data in these regions, the SDM was used to generate seasonal abundances estimates of Hector's and Māui dolphin throughout New Zealand. For Hector's dolphins, we showed that SDMs had poor transferability among populations, even when the environments were similar. There is, therefore, a potential risk that spatial transfer of a model will be unable to accurately predict distribution (e.g. for North Island Māui dolphin). Although we have focused on the transferability of models, this finding is also relevant to interpolation predictions made in the South Island. If dolphin populations use habitat differently, then models built from data collected on broad scales may represent cumulative distribution patterns, thus failing to accurately predict on a fine scale. Therefore we caution that SDMs should not be used to predict the distribution of Hector's dolphins outside the range where occurrence data are gathered, unless they are able to be validated with data from the novel habitat. If SDMs are used as an input to spatial management decisions, we recommend that they include locally derived data.

More generally, through this study we have added to the literature on SDM transferability, providing some evidence of the limitations of these approaches. A process by which models could reliably predict distributions to data poor areas would be of undeniable value (for a review, see Yates et al., 2018). The picture emerging from the literature on SDM transferability is mixed; currently it would be unwise to assume predictions are accurate in novel areas. When spatial transferability of SDMs is used in conservation planning, evidence of the accuracy of predicted distributions, where researchers validate models with independent data from the novel habitat, should be provided. If gaining such evidence is not possible, then the limitations of such an approach should be openly discussed and results interpreted with caution.

## AUTHOR CONTRIBUTIONS

**Steph Bennington:** Conceptualization (lead); data curation (lead); formal analysis (lead); funding acquisition (lead); investigation (lead); methodology (lead); project administration (lead); validation (equal); visualization (lead); writing – original draft (lead); writing – review and editing (lead). **Peter W. Dillingham:** Conceptualization (supporting); formal analysis (supporting); methodology (supporting); supervision (supporting); writing – review and editing (supporting). **Scott D. Bourke:** Data curation (supporting); methodology (supporting); writing – review and editing (supporting). **Stephen M. Dawson:** Conceptualization (supporting); supervision (supporting); writing – review and editing (supporting). **Elisabeth Slooten:** Conceptualization (supporting); supervision (supporting); writing – review and editing (supporting). **William J. Rayment:** Conceptualization (supporting); funding acquisition (supporting); methodology (supporting); supervision (lead); writing – review and editing (supporting).

## CONFLICT OF INTEREST STATEMENT

We have no conflict of interest to declare.

### OPEN RESEARCH BADGES

This article has earned an Open Data badge for making publicly available the digitally‐shareable data necessary to reproduce the reported results. The data is available at https://dataverse.harvard.edu/dataset.xhtml?persistentId=doi:10.7910/DVN/CY2MDN.

## Supporting information


Appendix S1.

## Data Availability

Data and R code, may be found at the following Dataverse repository: https://dataverse.harvard.edu/dataset.xhtml?persistentId=doi:10.7910/DVN/CY2MDN.
