# Supplementary material for: Testing spatial transferability of species distribution models reveals differing habitat preferences for an endangered delphinid (Cephalorhynchus hectori) in Aotearoa, New Zealand
Source: Ecol Evol. 2024 Jul 22;14(7):e70074. doi: 10.1002/ece3.70074 (PMC11262828; doi:10.1002/ece3.70074)

 **Appendices**

Appendix S1. Comparison of the environmental variability of covariates used in Species Distribution Modelling for Hector’s dolphin (*Cephalorhynchus hectori*) within and between locations along the southeast coast of the South Island: Banks Peninsula (n=433), Timaru (n=168) and Otago (n=238). Data are independent of dolphin presence and display the range of the environmental variables. Displayed are the median (solid line), the first and third quartile (upper and lower box) and the maximum non outliers (whiskers). Points are outliers. Here, we show the similarities and differences in the environmental range of covariates between each area.


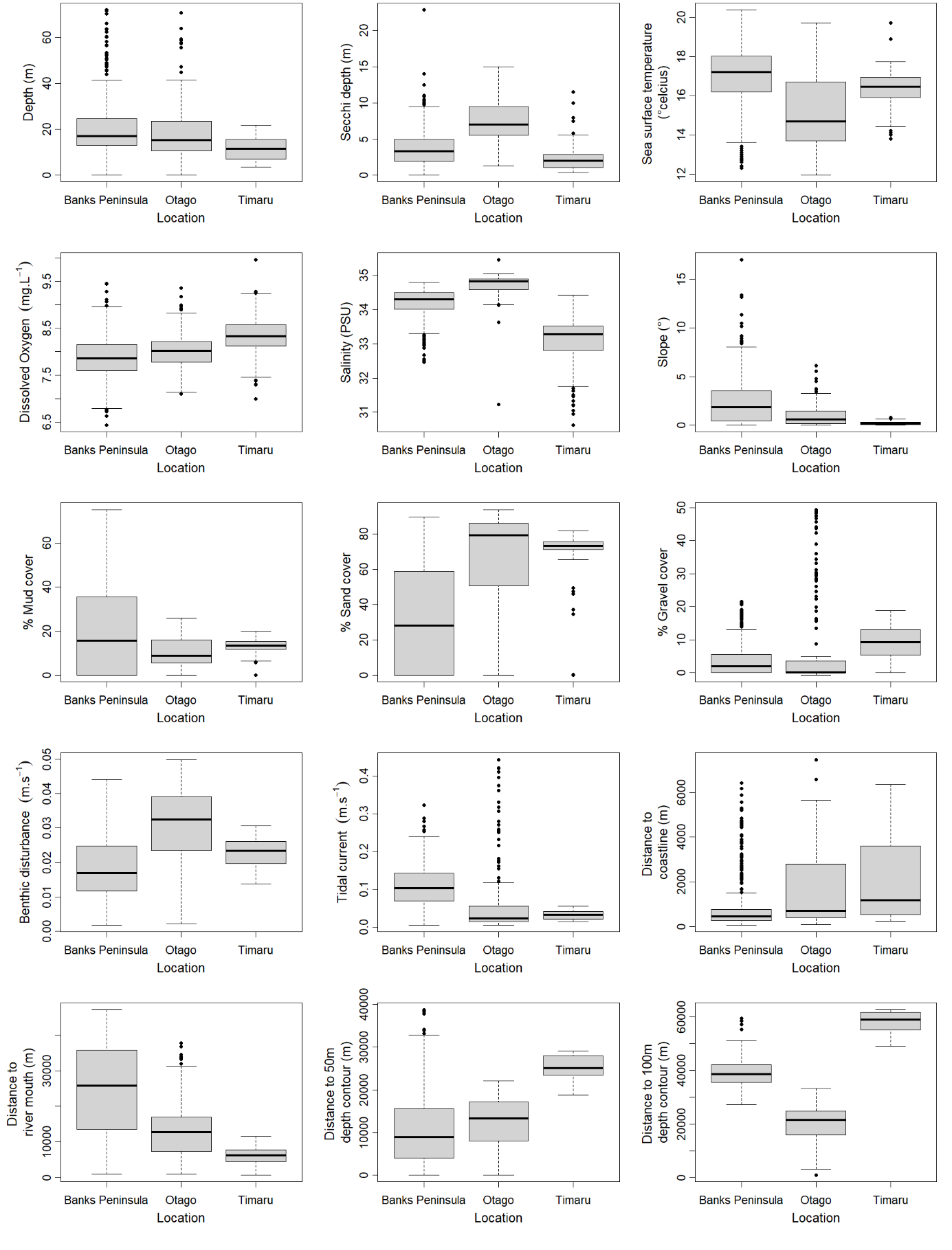

Supplement: Supplementary file 1 — Appendix S1. [file ECE3-14-e70074-s001.docx]
